# Quantitative Measures of Pure Ground-Glass Nodules from an Artificial Intelligence Software for Predicting Invasiveness of Pulmonary Adenocarcinoma on Low-Dose CT: A Multicenter Study

**DOI:** 10.3390/biomedicines13071600

**Published:** 2025-06-30

**Authors:** Yu Long, Yong Li, Yongji Zheng, Wei Lin, Haomiao Qing, Peng Zhou, Jieke Liu

**Affiliations:** 1 Department of Radiology, Sichuan Clinical Research Center for Cancer, Sichuan Cancer Hospital & Institute, Sichuan Cancer Center, University of Electronic Science and Technology of China, Chengdu 610041, China; longyu1301@foxmail.com (Y.L.); muzi5969@foxmail.com (Y.L.); qhm_pumc2004@163.com (H.Q.); penghyzhou@126.com (P.Z.); 2 Department of Radiology, Deyang People’s Hospital, Deyang 618000, China; zheng.yongji@foxmail.com; 3 Department of Radiology, Chengdu First People’s Hospital, Chengdu 610021, China; linwei_cfph@foxmail.com

**Keywords:** pulmonary adenocarcinoma, pure ground-glass nodule, low-dose CT, artificial intelligence, invasiveness

## Abstract

**Objectives**: Deep learning-based artificial intelligence (AI) tools have been gradually used to detect and segment pulmonary nodules in clinical practice. This study aimed to assess the diagnostic performance of quantitative measures derived from a commercially available AI software for predicting the invasiveness of pulmonary adenocarcinomas that manifested as pure ground-glass nodules (pGGNs) on low-dose CT (LDCT) in lung cancer screening. **Methods**: A total of 388 pGGNs were consecutively enrolled and divided into a training cohort (198 from center 1 between February 2019 and April 2022), testing cohort (99 from center 1 between April 2022 and March 2023), and external validation cohort (91 from centers 2 and 3 between January 2021 and August 2023). The automatically extracted quantitative measures included diameter, volume, attenuation, and mass. The diameter was also manually measured by radiologists. The agreement of diameter between AI and radiologists was evaluated by intra-class correlation coefficient (ICC) and Bland–Altman method. The diagnostic performance was evaluated by the area under curve (AUC) of receiver operating characteristic curve. **Results**: The ICCs of diameter between AI and radiologists were from 0.972 to 0.981 and Bland–Altman biases were from −1.9% to −2.3%. The mass showed the highest AUCs of 0.915 (0.867–0.950), 0.913 (0.840–0.960), and 0.893 (0.810–0.948) in the training, testing, and external validation cohorts, which were higher than those of diameters of radiologists and AI, volume, and attenuation (all *p* < 0.05). **Conclusions**: The automated measurement of pGGNs diameter using the AI software demonstrated comparable accuracy to that of radiologists on LDCT images. Among the quantitative measures of diameter, volume, attenuation, and mass, mass was the most optimal predictor of invasiveness in pulmonary adenocarcinomas on LDCT, which might be used to assist clinical decision of pGGNs during lung cancer screening.

## 1. Introduction

With the popularization of low-dose CT (LDCT) in the screening of lung cancer, there has been an increasing detection rate of subsolid nodules, which are more prevalent in females and non-smokers of Asian origin [1,2,3]. The persistent subsolid nodules are strongly associated with the spectrum of pulmonary adenocarcinomas [4,5]. In China, more than 90% of resected subsolid nodules turned out to be pulmonary adenocarcinomas [6,7]. Asia has the highest burden of lung cancer compared with America and Europe, and early diagnosis of lung cancer makes it curable and reduces mortality [8], resulting in the specific focus on early screening and diagnosis of lung cancers that manifest as subsolid nodules in Asian countries, especially in China, Japan, and Korea [9].

The subsolid nodules are radiologically categorized into pure ground-glass nodules (pGGNs) and part-solid nodules according to the absence or presence of solid component [10]. The presence of a solid component is pathologically associated with the invasiveness of pulmonary adenocarcinoma [11]. However, approximately 17% to 53% of pGGNs are pathologically proven to be invasive adenocarcinomas [12,13,14,15,16], which has a distinct prognosis compared with non-invasive adenocarcinomas, including adenocarcinoma in situ and minimally invasive adenocarcinomas. Although pGGNs tended to remain stable or show slow growth in the follow-up screening, it has also been found that over half of pGGNs progress during surveillance [17,18]. The invasiveness may be a hallmark of the initiation of growth. Previous studies showed that non-invasive adenocarcinomas were curable after limited resection, with a 10-year recurrence-free survival rate of 100% [19], and did not require systematic lymph node dissection or sampling [20]. While the invasive adenocarcinomas had a 5-year recurrence-free survival rate of 22% to 94%, depending on the differentiation grade [21,22], complete resection with lymph node dissection or sampling was necessary [23]. Therefore, invasiveness may inform the clinical decision of pGGNs during lung cancer screening. A conservative surveillance or limited resection without lymph node treatment can be adopted for non-invasive adenocarcinomas, while invasive adenocarcinomas require timely and more extensive surgical resection.

The diameter, determined by a two-dimensional (2D) caliper on CT images, is the most common quantitative measure in predicting the invasiveness of pulmonary adenocarcinomas [24,25,26], as it can be easily acquired by radiologists in clinical workflow and does not need additional efforts for segmentation. However, reproducible measurement of nodule diameter is still challenging due to inter- and intra-observer variability [27]. The developed computer-aided diagnosis and deep learning-based artificial intelligence (AI) tools have inherent advantages in reducing and eliminating this variability, and also provide more comprehensive three-dimensional (3D) quantitative measures such as volume, attenuation, and mass in addition to diameter [28]. However, which quantitative measure has the best performance in predicting the invasiveness of pGGNs remains controversial. Kim et al. found the mass had a higher area under curve (AUC) than volume but was not significantly higher than diameter and attenuation [15]. Han et al. found the AUCs of mass, volume, and diameter were higher than that of attenuation, while there was no significant difference among mass, volume, and diameter [14]. Others did not conduct direct comparisons [12,13]. Moreover, as all these studies used standard-dose CT rather than LDCT, the optimal quantitative measure to predict the invasiveness of pGGNs in the screening of lung cancer remained unestablished yet.

Therefore, this multicenter study aimed to assess the diagnostic performance of quantitative measures derived from a commercially available AI software for predicting the invasiveness of pulmonary adenocarcinoma in pGGNs on LDCT. The agreement in diameter between AI and radiologists was also evaluated.

## 2. Materials and Methods

### 2.1. Patients

This study was approved by the ethics committees of participating centers, and informed consent was waived due to its retrospective nature. The inclusion criteria were (a) patients with pGGNs detected and followed by LDCT in the screening of lung cancer; (b) persistent or progressive pGGNs during the follow-up period; (c) first treatment with surgical resection; and (d) pathologically diagnosed with pulmonary adenocarcinomas. The exclusion criteria were (a) part-solid nodules or solid nodules; (b) pathologically diagnosed with benign lesions; (c) the interval between the last LDCT scan and surgery over one month; and (d) poor image quality due to respiratory and movement artifacts.

**Figure 1:**
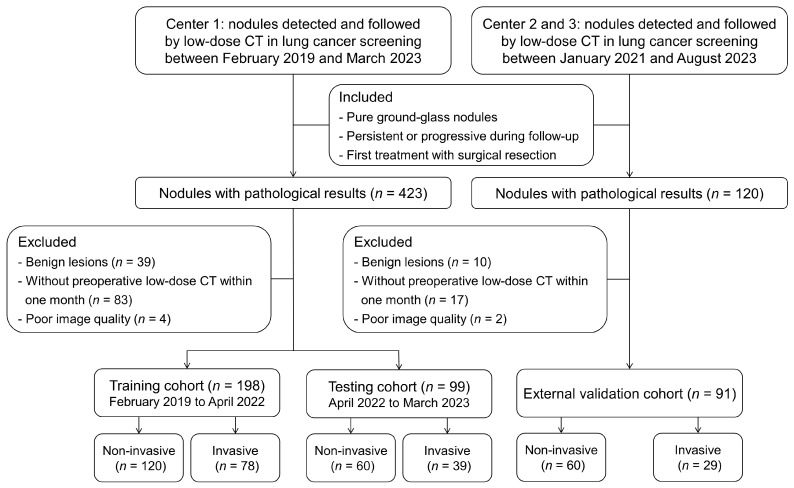
Patient selection workflow.

A total of 297 pGGNs between February 2019 and March 2023 were enrolled in center 1 (Sichuan Cancer Hospital) and divided into the training cohort (*n* = 198) and the testing cohort (*n* = 99) according to a ratio of 2:1 and the date of last LDCT scan. The external validation cohort (*n* = 91) was enrolled in center 2 (Deyang People’s Hospital) and center 3 (Chengdu First People’s Hospital) between January 2021 and August 2023. The patient selection workflow was illustrated in Figure 1.

### 2.2. Acquisition Parameters

The chest LDCT images were acquired using a 64-detector CT (Somatom Definition Flash, Siemens Healthcare, Forchheim, Germany) in center 1, a 96-detector CT (SOMATOM Force, Siemens Healthcare, Erlangen, Germany) in center 2, and a 64-detector CT (Somatom Definition Flash, Siemens Healthcare) and an 80-detector CT (uCT 780, United Imaging Healthcare, Shanghai, China) in center 3.

The acquisition parameters were as follows: tube voltage = 80 to 100 kV; tube current = 10 to 145 mAs; pitch = 1 to 1.5; collimation = 0.5 or 0.6 mm; rotation time = 0.25 to 0.5 s; field of view = 350 mm × 350 mm. Then, images were reconstructed using the following parameters: smooth reconstruction kernel (I30f for Somatom Definition Flash, Br40d for SOMATOM Force, and B_SOFE_B for uCT 780); slice thickness = 0.5 to 1 mm; no increment; matrix = 512 × 512. The mean and standard deviation of estimated effective dose were 0.66 mSv and 0.29 mSv.

### 2.3. Pathological Evaluation

The pathological diagnosis of pulmonary adenocarcinomas was obtained according to the 5th edition of World Health Organization (WHO) classification of thoracic tumors [29]. The pulmonary adenocarcinomas were classified as adenocarcinoma in situ, minimally invasive adenocarcinoma, and invasive adenocarcinoma. The adenocarcinoma in situ was defined as an adenocarcinoma of ≤3 cm with pure lepidic pattern. The minimally invasive adenocarcinoma was defined as an adenocarcinoma of ≤3 cm with lepidic predominant pattern and with an invasive component of ≤5 mm. The invasive adenocarcinoma was classified by the growth patterns using comprehensive histological subtypes of lepidic, acinar, papillary, micropapillary, solid, cribriform, and complex glandular. The percentage of each histological pattern was recorded in 5% increments.

### 2.4. Measure of Radiologists

Two radiologists (J.L. and H.Q., with 7 years and 12 years of experience) who were blinded to pathological diagnosis measured the diameter of pGGNs on axial LDCT images in lung window setting (level, −500 HU; width, 1500 HU). The measurement of diameter followed the Fleischner Society guideline [10]. The intra-class correlation coefficient (ICC) was used to evaluate the consistency of the diameter between two radiologists. The ICC > 0.75 indicated a good agreement. Then, the average of two radiologists was calculated for the following analysis.

### 2.5. Measures of AI

The uAI Discover Lung (United Imaging Intelligence, version R001), a commercially available AI software that was approved by the National Medical Products Administration (NMPA) of China in June 2021 (approval no. 20213210471), was used to automatically detect and segment pGGNs. Briefly, the uAI Discover Lung is composed of deep learning-based nodule detection and segmentation networks. The nodule detection network employs two cascade feature pyramid networks and a classification network (BasicNet), and the architecture of classification network contains four regular convolution-batch-normalized-ReLu blocks and a fully connected layer [30,31]. The nodule segmentation network is based on the VB-net, an extension of the V-Net that replaced the conventional down-sampling and up-sampling blocks with the bottleneck structure [32]. The uAI Discover Lung was trained and validated based on over 40,000 nodules; the average sensitivity (false positive range between 0 and 8) for nodule detection was 87.3%, and the average dice similarity coefficient was 91.5% for nodule segmentation [32].

**Figure 2:**
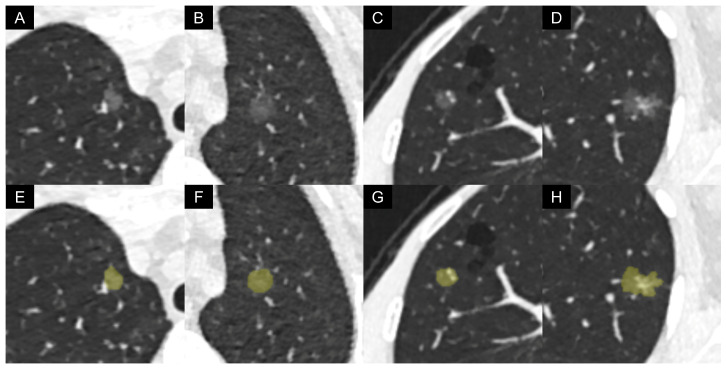
Representative low-dose CT images and artificial intelligence-based automatic segmentation results of pure ground-glass nodules: (**A**–**D**) original images; (**E**–**H**) segmentation results; (**A**,**E**) a 45-year-old female with non-invasive adenocarcinoma, with a vessel passing by the nodule; (**B**,**F**) a 74-year-old female with non-invasive adenocarcinoma, with a hazy boundary between the nodule margin and adjacent pulmonary parenchyma; (**C**,**G**) a 61-year-old female with invasive adenocarcinoma, with two dilated pulmonary vessels passing through the nodule; (**D**,**H**) a 47-year-old female with invasive adenocarcinoma, with irregular vascular dilation and vascular convergence within the nodule.

This AI software was integrated into the radiological diagnosis workflow side-by-side via connecting with the picture archiving and communication system (PACS). The output of the AI software included the maximal and minimum axial diameters, volume, attenuation, and mass of the nodule. The representative LDCT images and AI-based automatic segmentation results are presented in Figure 2. The diameter was the mean of the maximum axial diameter and the minimum axial diameter. The mass was calculated by the following equation [33]: Mass = Volume × (Attenuation + 1000)/1000. The units for diameter, volume, attenuation, and mass were mm, mm^3^, HU, and mg.

### 2.6. Statistical Analysis

Statistical analyses were conducted with Medcalc (version 18.2.1). The group differences were compared using the Mann–Whitney U-test for continuous variables and the Fisher’s exact test for categorical variables. The agreement in diameter between AI and radiologists was evaluated by the ICC and the Bland–Altman method [34]. The ICC > 0.75 indicated a good agreement. In the Bland–Altman analysis, a prespecified clinically acceptable limits of agreement (LOA) was set at −30% to 20% between AI and radiologists, which was a threshold for significant change [35]. The diagnostic performances of quantitative measures to predict the invasiveness of pulmonary adenocarcinomas were evaluated by the AUCs of receiver operating characteristic (ROC) curve analyses. The optimal cut-off was determined by Youden’s index in the training cohort and used to calculate sensitivity and specificity in all cohorts. The Delong test was conducted to compare the AUCs among quantitative measures [36]. Statistical significance was set at a two-sided *p* < 0.05.

## 3. Results

### 3.1. Demographic and Radiological Characteristics

The clinical characteristics and quantitative measures of pGGNs are summarized in Table 1. The percentages of invasive adenocarcinomas were 39.4% (78/198), 39.4% (39/99), and 31.9% (29/91) in the training, testing, and external validation cohorts.

The age in the invasive group was significantly higher than that in the non-invasive group in the training cohort (*p* < 0.001). No significant difference was found in gender (*p* = 0.347). The diameter of radiologists, diameter of AI, volume, attenuation, and mass of the invasive group were significantly larger than those of the non-invasive group (all *p* < 0.001).

Gender, age, and all quantitative measures showed no significant differences between the testing and training cohorts (all *p* > 0.05). The diameter of radiologists and diameter of AI in the external validation cohort were lower than those in the training cohort (*p* = 0.023 and *p* = 0.016). No significant differences in gender, age, volume, attenuation, and mass were found between the training and external validation cohorts (all *p* > 0.05).

### 3.2. Agreement of Diameter Between AI and Radiologists

**Figure 3:**
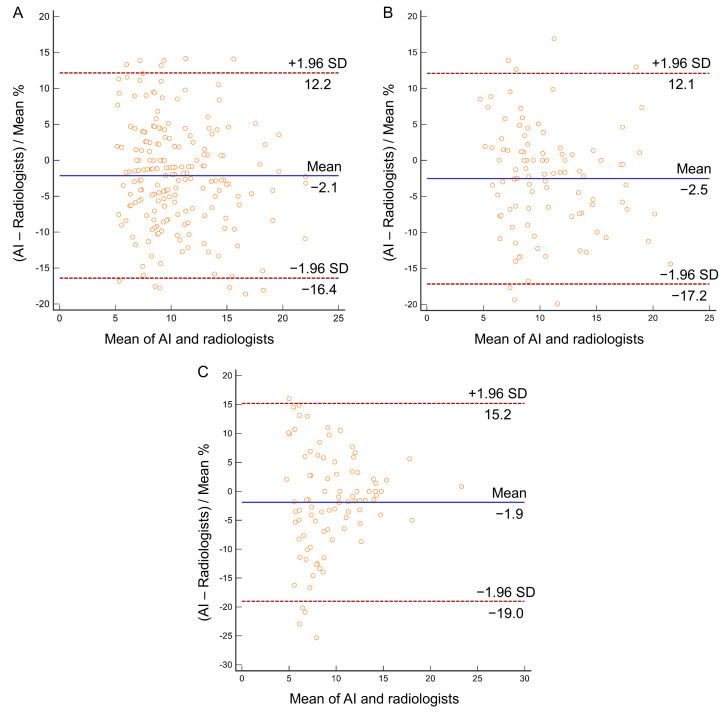
Bland–Altman plots for diameter of pure ground-glass nodules comparing the mean of AI and radiologists. (**A**) Training cohort; (**B**) testing cohort; (**C**) external validation cohort. AI, artificial intelligence.

The AI and radiologists yielded ICC values of 0.972 (0.959–0.980), 0.974 (0.957–0.984), and 0.981 (0.971–0.988) in the training, testing, and external validation cohorts. The Bland–Altman biases were −2.1% with a 95% LOA from −16.4% to 12.2%, −2.5% with a 95% LOA from −17.2% to 12.1%, and −1.9% with a 95% LOA from −19.0% to 15.2% in the training, testing, and external validation cohorts, respectively, falling within the prespecified clinically acceptable LOA. There were 12/198 (6.1%), 7/99 (7.1%), and 5/91 (5.5%) outliers in the training, testing, and external validation cohorts by inspection of the Bland–Altman plots (Figure 3).

### 3.3. Diagnostic Performances of Quantitative Measures

**Figure 4:**
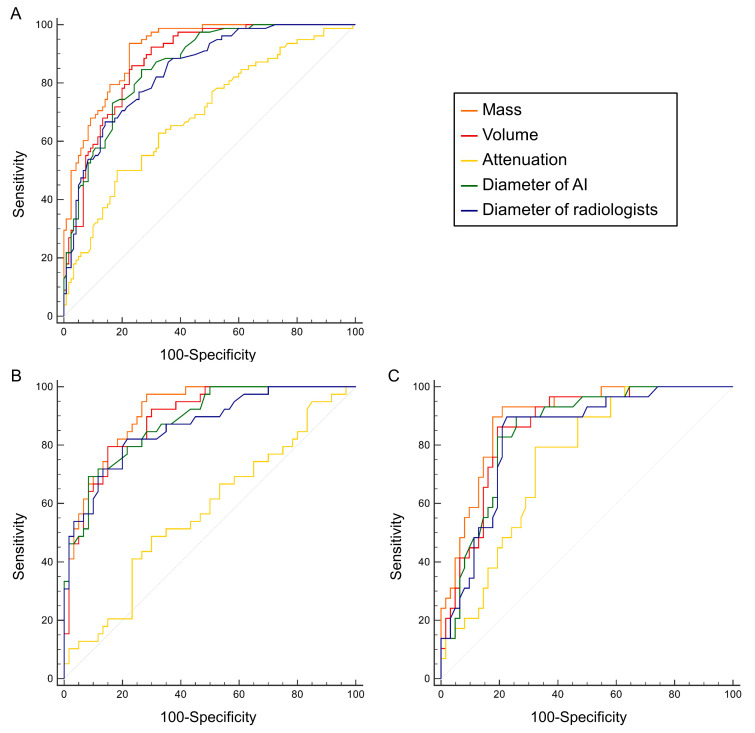
The receiver operating characteristic curves of quantitative measurements for differentiation between invasive and non-invasive adenocarcinomas in pure ground-glass nodules. (**A**) Training cohort; (**B**) testing cohort; (**C**) external validation cohort. AI, artificial intelligence.

The ROC curves of the measures of radiologists and AI are shown in Figure 4. The mass showed the highest AUC of 0.915 (0.867–0.950), 0.913 (0.840–0.960), and 0.893 (0.810–0.948) in the training, testing, and external validation cohorts among all quantitative measures.

The AUCs of diameter of radiologists, diameter of AI, volume, and attenuation were 0.844 (0.785–0.891), 0.861 (0.805–0.906), 0.877 (0.823–0.919), and 0.690 (0.620–0.754) in the training cohort; 0.865 (0.781–0.925), 0.882 (0.802–0.938), 0.890 (0.811–0.944), and 0.572 (0.469–0.671) in the testing cohort; and 0.831 (0.739–0.902), 0.847 (0.756–0.914), 0.860 (0.772–0.924), and 0.745 (0.643–0.831) in the external validation cohort. The detailed optimal cut-offs, sensitivities, and specificities are shown in Table 2.

The results of the DeLong test showed that the AUC of mass was significantly higher than that of the diameter of radiologists, diameter of AI, volume, and attenuation in the training, testing, and external validation cohorts (all *p* < 0.05, Table 3).

A comparison was also conducted between the diameter of radiologists and the diameter of AI. The AUC of the diameter of AI was significantly higher than that of the diameter of radiologists in the training cohort (Z = 2.211, *p* = 0.027), but no significant differences were found in the testing cohort (Z = 1.469, *p* = 0.142) or in the external cohort (Z = 1.210, *p* = 0.226).

### 3.4. Complementary Multivariable Analysis

As there was a significant group difference in age in the training cohort, we intended to integrate age and mass using multivariable logistic regression to further improve the diagnostic performance. However, age was not a significant predicting factor (*p* = 0.279), with an odds ratio (OR) of 1.019 (0.985–1.055) when combining with mass (OR = 1.010 [1.007–1.014], *p* < 0.001).

## 4. Discussion

This multicenter study confirmed the feasibility of automatically measuring the diameter of pGGNs using AI software on LDCT. Among the quantitative measures of diameter, volume, attenuation, and mass, mass was found to be the most optimal predictor of invasiveness in pulmonary adenocarcinomas that manifested as pGGNs on LDCT.

Recent studies have shown that the maximal diameter of pGGN and the maximal diameter of the solid component measured by AI-based tools were comparable with the manual measurement [37,38]. Here, we initially focused on the mean diameter, which was required for the Fleischner Society guideline and the Lung CT Screening Reporting & Data System (Lung-RADS) [10,39]. It was found that the diameter measured by AI software was slightly smaller than that measured by radiologists in pGGNs, which was inconsistent with previous findings [38]. The using of LDCT and a smooth kernel rather than standard-dose CT and a sharp kernel might contribute to this discrepancy. With the reduction in radiation dose and the use of a soft reconstruction algorithm, the boundary between the nodule margin and adjacent pulmonary parenchyma might be hazier, resulting in the underestimate of diameter in pGGNs by AI software. In ICC and Bland–Altman analyses, the diameters of AI and radiologists showed strong agreement and excellent repeatability, indicating that the AI software might be an acceptable alternative for radiologists in measuring the diameter of pGGNs.

In the era of computer-aided diagnosis, pulmonary nodule detection and segmentation are usually integrated into a post-processing workstation as a modular function. Radiologists firstly need to transfer the targeted images from the scanner or PACS to the workstation and then conduct the postprocessing one by one. Moreover, these computer-aided diagnosis tools often need manual intervention for nodule segmentation, such as clicking on the nodule to initiate the segmentation and dragging the mouse to modify the boundary. In China, the radiologists in tertiary hospitals are burdened with substantial workloads, often required to interpret and report on 80 to 100 CT scans daily [40]. Under the circumstances, manual or semi-automatic segmentation approaches are challenging to implement in routine clinical practice. The deep learning-based AI tools, by contrast, automatically executing side-by-side with PACS and providing a user-friendly interface with automatic measurement of nodules, increase the efficiency of reading the images [28]. Currently, the Food and Drug Administration (FDA) has gradually approved AI-based tools in radiology, which provide related image information to radiologists and do not generate diagnoses directly [41]. In the context of a shortage of radiologists, the Chinese Radiology Society (CSR) has established the committee of AI to facilitate the implementation of AI in medical imaging applications [40]. It can be expected that more and more AI-based tools will fulfill the tasks of auxiliary diagnosis in radiology, comparable to previous computer-aided diagnosis tools.

A previous study showed the AI-derived maximal diameter of pGGN was correlated with invasiveness with an AUC of 0.833 [38], and a similar result was found in the mean diameter in the present study. Furthermore, we investigated several 3D quantitative measures and found the diagnostic performance of mass was superior to that of diameter, volume, and attenuation. The nodule mass showed better ability to detect growth of subsolid nodules early and less measurement variability than diameter and volume [42]. However, in terms of predicting the invasiveness of pGGNs, previous studies reported inconsistent findings, which might be due to the use of different reconstruction kernels. Kim et al. and Han et al. used sharp kernels and found the mass was not superior to diameter or volume in pGGNs [14,15]. Wang et al. evaluated the diagnostic performances of quantitative measures to predict the invasiveness of part-solid nodules using a medium-sharp kernel and found the mass was superior to diameter and volume [43]. It is known that the reconstruction kernel can significantly affect the attenuation [44,45,46]. Measuring the attenuation on sharpened images may overestimate its value [10], which further leads to erroneous quantification of the mass. Unlike standard-dose CT, LDCT images are usually reconstructed with smooth kernels to reduce the noise and improve the visibility of nodule details [27]. Our results suggest the nodule mass underlying the smooth reconstruction algorithm was the optimal quantitative measure to predict the invasiveness of pulmonary adenocarcinomas, which might assist radiologists and thoracic surgeons in the management of pGGNs during lung cancer screening.

Consistent with prior studies that utilized manual [12,13,14,42] or semi-automatic segmentation [15] methods to measure the mass of pGGNs, the AI software employed in this study did not exclude pulmonary vessels within pGGNs. Compared with the exclusion of vessels, the inclusion of vessels in the measurement of pGGNs indeed compromises the accuracy of nodule metrics. Specifically, it leads to an overestimation of nodule volume, attenuation, and mass, thereby resulting in an increased probability of invasiveness. However, from the perspective of radiological diagnosis, the amount and complexity of pulmonary vessels within subsolid nodules were highly associated with the invasiveness [47,48]. Therefore, excluding pulmonary vessels might potentially result in the loss of critical information for predicting invasiveness. Some researchers have developed automatic segmentation algorithms to exclude vessels within subsolid nodules [49,50]. To our knowledge, such algorithms have not yet been incorporated into the AI software currently employed in clinical practice. Future development of AI software should integrate automatic segmentation algorithms capable of excluding pulmonary vessels within nodules. This advancement would facilitate further validation studies to determine whether excluding or including vessels is more advantageous for predicting the invasiveness of pGGNs.

This study had several limitations. The first is the retrospective nature of the study, which only includes persistent and progressive pGGNs detected and followed in lung cancer screening that underwent surgical resection; this limited the sample size for other lung diseases and increased the risk of inclusion of more invasive adenocarcinomas. It is essential to expand the inclusion criteria to encompass pGGNs that are transient and do not undergo surgical resection in future work. From a technical perspective, this comprehensive approach will enable a more robust investigation into the relationship between pGGNs and other lung diseases and their staging types. Second, excluding benign lesions also constituted selection bias. Although persistent subsolid are strongly associated with the spectrum of pulmonary adenocarcinomas [4,5], and over 90% surgically resected pGGNs were pulmonary adenocarcinomas in China [6], which was similar to this study (90.8% in center 1; 91.7% in centers 2 and 3), a differential diagnosis between benign and malignant persistent pGGNs should also be considered in future study with a sufficient sample size of the benign group. Third, the use of a proprietary and non-open-source AI software limited the generalizability of our results. In addition, quantitative measures derived from different AI software platforms may exhibit variability. Further studies employing multiple and open-source AI software programs are needed to validate our findings.

## 5. Conclusions

In conclusion, automated measurement of the diameter of pGGNs on LDCT images using the AI software demonstrated comparable accuracy to that achieved by radiologists. Among the quantitative measures of diameter, volume, attenuation, and mass, mass was the most optimal predictor of invasiveness in pulmonary adenocarcinomas on LDCT. This finding suggests that the mass could be used to guide the clinical decision-making regarding the management of pGGNs during lung cancer screening. Future development of AI software should incorporate automatic segmentation algorithms capable of excluding pulmonary vessels within pGGNs. Prospective longitudinal studies employing multiple and open-source AI systems are warranted to validate our findings. Additionally, elucidating the predictive capacity of the AI-derived quantitative measures for long-term prognosis of early-stage pulmonary adenocarcinomas will further enhance their clinical application value.

## Figures and Tables

**Table 1:** Characteristics of pure ground-glass nodules in the training, testing, and external validation cohorts.

Characteristics	Training Cohort (*n* = 198)			Testing Cohort (*n* = 99)			External Validation Cohort (*n* = 91)		
	Non-Invasive (*n* = 120)	Invasive (*n* = 78)	*p* ^a^	Non-Invasive (*n* = 60)	Invasive (*n* = 39)	*p* ^b^	Non-Invasive (*n* = 62)	Invasive (*n* = 29)	*p* ^b^
Gender			0.347			0.792			>0.999
Female	79	57		43	23		43	19	
Male	41	21		17	16		19	10	
Age (years)	49.8 (12.0)	58.1 (10.4)	<0.001	50.2 (11.8)	58.0 (9.3)	0.757	50.3 (11.7)	59.0 (12.9)	0.830
Diameter of radiologists (mm)	8.9 (2.6)	13.2 (3.8)	<0.001	8.7 (2.4)	13.9 (4.0)	0.867	8.3 (2.6)	12.1 (3.4)	0.023
Diameter of AI (mm)	8.6 (2.4)	12.9 (3.5)	<0.001	8.5 (2.2)	13.6 (3.8)	>0.999	8.0 (2.6)	12.2 (3.4)	0.016
Volume (mm^3^)	375.0 (404.3)	1213.1 (874.0)	<0.001	350.3 (348.6)	1425.7 (1266.8)	0.936	410.4 (461.4)	1314.0 (1432.5)	0.724
Attenuation (HU)	−652.1 (76.5)	−592.4 (90.6)	<0.001	−645.8 (76.6)	−622.2 (92.2)	0.379	−658.3 (76.2)	−591.4 (75.6)	0.484
Mass (mg)	118.9 (104.6)	485.7 (376.2)	<0.001	119.3 (105.0)	489.0 (388.1)	0.997	131.3 (127.4)	528.0 (511.2)	0.570

^a^ Comparisons between non-invasive and invasive group. ^b^ Compared with the training cohort. Continuous variables are means with standard deviations in parentheses. AI, artificial intelligence.

**Table 2:** Diagnostic performances of quantitative measurements for differentiation between non-mucinous invasive and non-invasive adenocarcinomas in pure ground-glass nodules.

Characteristics	AUC (95% CI)	Sensitivity (95% CI)	Specificity (95% CI)	Optimal Cut-Off
Training cohort				
Diameter of radiologists	0.844 (0.785–0.891)	0.667 (0.551–0.769)	0.858 (0.783–0.915)	>11.1 mm
Diameter of AI	0.861 (0.805–0.906)	0.846 (0.747–0.918)	0.733 (0.645–0.810)	>9.4 mm
Volume	0.877 (0.823–0.919)	0.859 (0.726–0.927)	0.767 (0.681–0.839)	>410.1 mm^3^
Attenuation	0.690 (0.620–0.754)	0.500 (0.385–0.615)	0.837 (0.736–0.881)	>−590.6 HU
Mass	0.915 (0.867–0.950)	0.936 (0.857–0.979)	0.775 (0.690–0.846)	>144.5 mg
Testing cohort				
Diameter of radiologists	0.865 (0.781–0.925)	0.692 (0.524–0.830)	0.867 (0.754–0.941)	NA
Diameter of AI	0.882 (0.802–0.938)	0.795 (0.635–0.907)	0.750 (0.621–0.853)	NA
Volume	0.890 (0.811–0.944)	0.821 (0.665–0.925)	0.733 (0.603–0.839)	NA
Attenuation	0.572 (0.469–0.671)	0.256 (0.130–0.421)	0.767 (0.640–0.866)	NA
Mass	0.913 (0.840–0.960)	0.897 (0.758–0.971)	0.750 (0.621–0.853)	NA
External validation cohort				
Diameter of radiologists	0.831 (0.739–0.902)	0.621 (0.423–0.793)	0.806 (0.686–0.896)	NA
Diameter of AI	0.847 (0.756–0.914)	0.828 (0.642–0.942)	0.758 (0.633–0.858)	NA
Volume	0.860 (0.772–0.924)	0.862 (0.683–0.961)	0.742 (0.615–0.845)	NA
Attenuation	0.745 (0.643–0.831)	0.448 (0.264–0.643)	0.790 (0.668–0.883)	NA
Mass	0.893 (0.810–0.948)	0.897 (0.726–0.978)	0.823 (0.705–0.908)	NA

AUC, area under curve; CI, confidence interval; AI, artificial intelligence; NA, not applicable.

**Table 3:** Comparisons of area under the curves between mass and other quantitative measurements.

Characteristics	Training Cohort		Testing Cohort		External Validation Cohort	
	Z	*p*	Z	*p*	Z	*p*
Diameter of radiologists	4.710	<0.001	2.212	0.027	3.017	0.003
Diameter of AI	4.387	<0.001	1.998	0.046	3.177	0.002
Volume	4.636	<0.001	2.060	0.039	2.709	0.007
Attenuation	5.062	<0.001	5.009	<0.001	2.430	0.015

AI, artificial intelligence.

## Data Availability

The data presented in this study are available on request from the corresponding author. The data are not publicly available due to the institutional privacy policies.

## Abbreviations

2D	Two-dimensional
3D	Three-dimensional
AI	Artificial intelligence
AUC	Area under curve
CSR	Chinese Radiology Society
FDA	Food and Drug Administration
ICC	Intra-class correlation coefficient
LDCT	Low-dose CT
LOA	Limits of agreement
Lung-RADS	Lung CT Screening Reporting & Data System
NMPA	National Medical Products Administration
OR	Odds ratio
PACS	Picture archiving and communication system
pGGN	Pure ground-glass nodule
ROC	Receiver operating characteristic
WHO	World Health Organization
